# Public Policy Research—Born in the USA, at Home in the World?

**DOI:** 10.1007/s11615-022-00396-5

**Published:** 2022-05-02

**Authors:** Nils C. Bandelow, Nicole Herweg, Johanna Hornung, Reimut Zohlnhöfer

**Affiliations:** 1 Institute of Comparative Politics and Public Policy (CoPPP), Technische Universität Braunschweig, Bienroder Weg 97, 38106 Braunschweig, Germany; 2 Ruprecht-Karls-Universität Heidelberg, Heidelberg, Germany

**Keywords:** Policy studies, Policy analysis, Theories of the policy process, Policy sciences, Comparative politics, Politikfeldanalyse, Staatstätigkeitsforschung, Theorien politischer Prozessforschung, Öffentliche Politik, Vergleichende Regierungslehre

## Abstract

Public policy emerged as an academic subfield in the United States after the second World War. The theoretical foundations of the discipline are essentially based on analyses of Anglo-Saxon policies and politics and were originally aimed at providing knowledge for the policy process of pluralistic democracies. Given the increasing transfer of the subject and related approaches to other countries, it is necessary to clarify how suitable theories, goals, and methods of policy research are applied in other contexts. What needs to be considered when transferring theories of the policy process, and what can be learned from existing applications of the various approaches in different settings? The compilation of contributions on selected theoretical public policy lenses and their transfer to other countries and regions provides a nuanced answer to these questions.

## Abstract (de)

Policy-Forschung ist als wissenschaftliche Teildisziplin nach dem Zweiten Weltkrieg in den USA entstanden. Das theoretische Fundament des Fachs basiert wesentlich auf Analysen angelsächsischer Politik und war ursprünglich darauf ausgerichtet, Wissen für politische Prozesse in pluralistischen Demokratien bereitzustellen. Angesichts der zunehmenden internationalen Ausbreitung des Fachs ist zu klären, inwiefern die Theorien, Ziele und Methoden für Anwendungen in anderen politischen Systemen geeignet sind. Was muss jeweils bei einer Übertragung von Theorien der Policy-Forschung beachtet werden, und was lässt sich aus den bisherigen Anwendungen der verschiedenen Ansätze in unterschiedlichen Kontexten lernen? Die Zusammenstellung von Beiträgen zu ausgewählten Ansätzen und deren Transfer in andere Länder und Regionen gibt eine differenzierte Antwort auf diese Fragen.

## Introduction: Public Policy as an Academic Subfield

Public policy as a scientific subfield emerged after World War II, with strong roots in the United States. These U.S. roots shaped not only the empirical focus of most of the field’s early studies but also the theoretical perspectives and normative goals of the discipline. With the foundation of specialized journals such as *Policy Sciences* in 1970 and the *Policy Studies Journal* in 1972, the idea of gaining both knowledge of the policy process and knowledge for the policy process (Lasswell 1970) was merged under varying disciplinary terms, including “policy sciences,” “policy analysis,” “policy studies,” “policy research,” and “public policy.” The journals were accompanied by textbooks, academic organizations, degree programs, and conferences to establish a community of scholars using a similar terminology and common theoretical perspectives to understand and improve the policy process. Despite rapid success of the subject in the Anglo-Saxon world, public policy took decades to establish itself in other countries and continents. In Germany, public policy was first established not as an academic subdiscipline but as a way to improve policy-making in the late 1960s and early 1970s (Janning and Toens 2008). Several terms, such as “Policy Analyse” (Windhoff-Héritier 1987), “Staatstätigkeitsforschung” (Schmidt 1988), “Politikfeldanalyse” (Schubert 1991), and “Policy-Forschung” (Wenzelburger and Zohlnhöfer 2015), have been used to translate both the terms and the perspectives of the field. The variety of terms is an expression of the still unfinished effort to find a common view of the objects and objectives of the subdiscipline. A particular challenge lies in public policy research being confronted with the particularities of different political systems, norms, actors, traditions, and administrative and academic structures.

The *Politische Vierteljahresschrift* (PVS; German Political Science Quarterly) has used its special issues in particular to contribute to the transfer and development of public policy in Germany. With a focus on comparative perspectives, Manfred G. Schmidt compiled a special issue in 1988 of contributions by scholars from mostly German-speaking countries. This 19th *Sonderheft* of the PVS focused on institutional explanations for analyzing conflicts and negotiations in the policy process, with an explicit reference to Anglo-Saxon policy analysis. Another PVS *Sonderheft* called “Policy-Analyse” appeared only 5 years later, compiled by Adrienne Héritier (1993). This publication included both German and international perspectives to discuss several criticisms and developments of policy research that were seen as elements contrary to the “textbook policy process” (Nakamura 1987). Beyond contributions by several other renowned scholars, such as Peter deLeon, Frank Fischer, B. Guy Peters, Michael Howlett, Giandomenico Majone, Renate Mayntz, and Fritz W. Scharpf, it included a first presentation of the Advocacy Coalition Framework by Paul A. Sabatier translated into German (Sabatier 1993).

Since then and throughout the last years, policy research as a subdiscipline has been gaining greater relevance in Germany (Jann 2009), including visibility through German and German-language textbooks on its central approaches (Knill and Tosun 2020; Sager et al. 2017; Schubert and Bandelow 2014; Wenzelburger and Zohlnhöfer 2015). One main research interest lies in detecting the reasons for policy change and stability. However, when theoretical perspectives are exported from their original context, it is necessary to ensure that the key explanation on which they focus is adequately adapted. It is therefore worth asking how far the perspectives of policy research can be expanded to new countries and regions and what needs to be considered when applying them across different settings.

At the time of the last PVS special issue on policy research in 1993, the field had only just begun to systematically exchange theories and perspectives among national communities. In the meantime, we have a well-established international community that regularly exchanges theories, methods, and results. The community uses not only journals but also edited books to define the state of research on a regular basis. Most influential, at least with regard to policy process theories, is *Theories of the Policy Process*, the first edition of which was compiled by Paul Sabatier in 1999 and which has been co-edited by Christopher Weible since its third edition. The established theories and frameworks included in this book (the fifth edition of which is currently being prepared) are applied to many cases. As with public policy as an academic field, the theories included in these compilations all have strong roots in the United States, most leading scholars are based in rich democracies, and the original applications usually come from North America. However, most theories have spread step by step to other regions. Moreover, literature reviews confirm strong growth in applications of the perspectives, even beyond the boundaries of their initial origins (Jones et al. 2016; Kuhlmann and van der Heijden 2018; Pierce et al. 2017).

These observations have led to the question of the respective theories’ regional scope. In which countries do applications of these theoretical lenses exist, how many are there, which aspects are taken up, and how well do these theories travel? This special issue is particularly interested in these questions and explores issues, applications, and foci of the respective research communities in regions that were not initially major application areas. This interest not only encompasses systematically recording the assumptions and prerequisites in the application of the frameworks and examining them as to their transferability to specific political systems and policy fields, but it also means going beyond the academic debate to assess the relevance of the theories for solving societal problems and evaluating the relationship between scientific evidence and political decisions (Mayntz and Scharpf 1975).

The analytical focus of this special issue is on the opportunities and challenges associated with transferring theories to other regions. One may start with the foundations of a theory to discuss how far these assumptions go in other regions and countries. The United States is a large country with an established representative democracy, strong presidential federalism, pluralistic interest intermediation, and specific political and scientific values and cultures. Every theory transfer thus faces the challenge of examining possible obstacles and adaptation requirements. Which theories are appropriate in states without presidentialism, federalism, and interest group pluralism, let alone without an established democracy? What should be considered when studying public policy in small or fragile states?

To take on this endeavor, the contributions to this special issue each treat one theoretical perspective of current research. Thereby, they cover the most prominent and some emerging perspectives on policy research, namely the Advocacy Coalition Framework (Jenkins-Smith et al. 2018), the Multiple Streams Framework (Herweg et al. 2018), the social identity perspective of the Programmatic Action Framework (Bandelow et al. 2021; Hassenteufel and Genieys 2021), the Narrative Policy Framework (Shanahan et al. 2018), the Punctuated Equilibrium Theory (Baumgartner et al. 2018), the German-style “Vergleichende Staatstätigkeitsforschung” (Schmidt 1993), policy feedback and responsiveness theories (Béland and Schlager 2019), policy styles (Howlett and Tosun 2019; Richardson 1982), and evidence-based policy-making (Cairney 2016). The contributions examine the respective theories’ traveling capacity to other geographic regions, including those with nonrepresentative democratic political systems.

## Contributions to this Special Issue

About 30 years after its German presentation by Paul Sabatier (1993), the Advocacy Coalition Framework (ACF) has become one of the mainstream lenses of policy research. The ACF has informed several hundreds of empirical studies all over the world. However, there is still an overrepresentation of ACF studies in North America (Pierce et al. 2017). Over the last decades, Europe has also established itself as a second highly relevant area for ACF studies (Bandelow et al. 2019; Hornung and Bandelow 2021; Nohrstedt and Olofsson 2016). Applying the ACF to Europe confronts this framework with stronger and more diverse parties, smaller countries, and different cultures. However, most North American and European countries are relatively wealthy and have a more or less established democratic culture. Applications in Asia came much later but have been increasing in recent years (Li and Weible 2021; Nam et al. 2022; Ohno et al. 2021). Applying the ACF to Asia confronts the framework with a diversity of political systems and cultures. The challenge becomes even greater when the ACF seeks to analyze policy processes in African countries. In addition to political and cultural differences, applications in Africa need to consider socioeconomic conditions and sometimes fragile statehood. What does this mean for the transferability of the ACF, and perhaps policy research in general, to African cases? To approach this question, it is first necessary to take stock of applications to date. The first contribution to this special issue by *Alex Osei-Kojo, Karin Ingold, and Christopher M. Weible* (2022) presents a systematic literature review of the ACF in Africa. The authors identify 27 ACF applications to African cases, with a specific focus on their geographic distribution, themes, and theoretical and methodological foci. The review shows that ACF applications to Africa are growing and revolve especially around environmental and energy issues, following the global trend. Most of the articles study advocacy coalitions and policy change, while the study of policy-oriented learning is underrepresented. The authors furthermore identify studies that combine the ACF with other theoretical perspectives such as the Narrative Policy Framework and comparative studies focusing on multiple policy subsystems. Methodologically, the identified applications follow the classic ACF focus on single-case studies and qualitative approaches. The authors find that the ACF is portable to the African context but call for greater consideration of the second-generational, deeper questions of the ACF about policy processes.

The special issue proceeds with a systematic discussion of the conceptual transferability of the Multiple Streams Framework (MSF) to nondemocratic forms of government. This perspective is based on the garbage-can model of organizational choice and was introduced to policy research by John Kingdon (2003), based on research in the United States. In the meantime, the framework has become a prominent perspective for analyzing the policy process, often with a particular focus on agenda-setting in democracies (Béland and Howlett 2016; Herweg 2016; Herweg et al. 2018; Saurugger and Terpan 2016; Staff 2020; Zahariadis 2016; Zohlnhöfer et al. 2016). In their contribution to this special issue, *Nicole Herweg, Nikolaos Zahariadis, and Reimut Zohlnhöfer* (2022) reflect on whether and how the MSF can be applied to policy processes in autocracies, and the authors discuss the implications the particularities of these systems may have for the MSF. Even though the main idea that political decision-making occurs under ambiguity and time constraints is accurate independently of political systems, there are several challenges that policy-making in autocracies might pose for the application of the MSF. Restricted societal pluralism, limited media freedom, centralized political decision-making, and lack of contested elections could necessitate substantial adaptations to the framework. Nonetheless, the conceptual analysis reveals that the MSF may travel surprisingly well to nondemocratic systems with comparatively few adaptations. For example, the authors hypothesize that, in autocracies, conditions are more likely to be framed as problems when they regard issues of particular ideological importance to the leader and the leading elite. Similarly, conditions are less likely to become perceived as problems when they can be interpreted as signifying a government failure. The policy stream in autocracies is likely to be made up of a rather integrated policy community, while the most important criterion a policy idea has to fulfill is the approval of the leadership. Similarly, while the autocratic leadership is likely to dominate the political stream, the authors argue that the other elements of that stream—including the national mood—also remain relevant in autocratic regimes. Finally, the concepts of policy window and coupling can also be transferred to autocracies with some modifications. For example, the authors argue that rather than concerns about reelection, regime stability may trigger the opening of a problem window.

The third contribution deals with the social identities in the policy process (SIPP) perspective. This approach, which is fairly well-established in social psychology, follows the idea that each individual, including policy-makers, feels a sense of belonging to social groups that are characterized by a shared social identity. This includes regional identity, organizational identity, sectoral identity, demographic identity, and informal identity (Hornung et al. 2019). The drivers of policy preferences, and thereby policy change, can be explained by group membership and the salience of the respective social identities at a given time. The social identity approach itself emerged in Anglo-Saxon research (Haslam 2001; Hogg 2016). In public policy, it became a major tool of the Programmatic Action Framework (PAF), an approach that has its origins in French elite research (Bandelow and Hornung 2020; Hassenteufel et al. 2010; Hornung 2022). Even though the psychological core of the SIPP perspective can be argued to be universal, political conditions may influence the relevance and salience of certain identities. To assess the travel capacity of this approach, *Johanna Hornung, Ilana Schröder, and Nils C. Bandelow* (2022) conduct a comparative analysis of the social identities of civil servants in the Russian Federation Ministry of Health and the Ministry of Natural Resources and Ecology. The analysis indicates that in a more hierarchical and authoritarian political system like Russia, organizational identity and thereby party membership seem to be less relevant for influential positions in ministries. In contrast, informal social identities and networks have a greater influence. The relevance of identities, however, differs with regard to the importance of the ministry’s sector for the government. Moreover, the two ministries show a strong representation of thematic experts, leading to the formulation of professional identity as another distinct identity type. In line with the reflection of the MSF contribution, the application of SIPP to Russia shows that while the theoretical idea is transferable to another political context, access to data on policy actors and their networks is limited, making it methodologically difficult to apply the approach in less transparent political systems.

The Narrative Policy Framework (NPF) is a relatively new theory of the policy process that has concomitantly grown in its application over the last decade (Jones and McBeth 2010; Shanahan et al. 2018). The premise of the NPF is that narratives influence policy formation, adoption, implementation, and evaluation. Political actors and strategists understand that how a story is constructed is critical to policy success across all stages of the policy process. The NPF has asserted that the framework is readily transportable to varying policy and political contexts (Smith-Walter and Jones 2020). *Caroline Schlaufer, Johanna Künzler, Michael D. Jones, and Elizabeth A. Shanahan* (2022) contribute to this special issue by examining how extensively and in which contexts the NPF has already been used. Through a comprehensive review of extant NPF studies, they identify 157 scholarly applications of the framework up to July 2021. The objective of this review is to map the new territories that the NPF has explored outside of its original U.S. context. Importantly, this review examines not only geographic and policy domain variation but also data sources, methodological applications, levels of analysis, and the framework’s use in tandem with other theories of the policy process (e.g., ACF, MSF, Institutional Analysis and Development Framework). The authors find that while most studies are concentrated in the U.S. context, the expansion to other geographies such as Europe is increasing. Interestingly, the extent to which the NPF may be applied to authoritarian regimes is currently being tested, with recent work in Russia specifically (Schlaufer et al. 2021; Uldanov et al. 2021). Similarly, while most policies that apply the NPF remain centered on environmental policies, there is recent expansion to other policy domains (e.g., education). The authors also find that most NPF scholarship employs content analyses of media at the mesolevel of analysis, presumably because these data are easily captured in the public domain and are free. In sum, the authors find that the portability of the NPF has not reached its potential, given that the focus of the early years of NPF scholarship was devoted to establishing the validity of the framework itself.

The Punctuated Equilibrium Theory (PET), the fifth contribution to this special issue, has its roots in explaining issue attention and agenda-setting in American politics (Baumgartner and Jones 1993). Since the early 2000s, scholars have used the methodology of this approach to collect data for media and government attention on different issues all over the world. The Comparative Agendas Project (CAP) provides a platform to present these data from more than 20 countries (Baumgartner et al. 2019). The broad international success of the PET contributed to the development of a stronger focus in comparative politics, addressing venues and issue responsiveness of different political systems (Mortensen and Green-Pedersen 2014). Based on this perspective, *Daniela Beyer, Christian Breunig, Christoffer Green-Pedersen, and Jonathan Klüser* (2022) present a case study on the attention of parties, members of parliament, and governments for digitalization since 1990 under the specific conditions of the German federal consensus democracy. The study shows the belated interest in the issue by the government and political parties and the change of perspectives from an infrastructure issue to a central subject of general economic policy. Further research should focus not only on the role of courts for agenda-setting in Germany (Töller 2021) but also on the relationship between digitization and various other policy areas, such as health (Bogumil-Uçan and Klenk 2021).

Most lenses of policy research combine theoretical ideas with a specific set of methods. This is also true for the perspective made prominent by Manfred G. Schmidt and often referred to in Germany as the “Heidelberger Schule der Vergleichenden Staatstätigkeitsforschung” (Zohlnhöfer 2008): It assumes that various characteristics of states (or, more rarely, other entities such as regions) jointly contribute to explaining policies. Such characteristics originally included socioeconomic conditions, the partisan composition of cabinets, the power of organized interests, the number of veto players, globalization and Europeanization, and policy inheritance (Obinger et al. 2014; Schmidt 1993; Zohlnhöfer and Obinger 2005; Zohlnhöfer et al. 2008). Most recently, various other conditions have supplemented them, each of which was oriented toward specific theories (not only from policy research), for example, the varieties of capitalism (Höpner 2009; Schröder 2013). The relationship between these explanatory variables and policy outcomes (and, less frequently, outputs) usually involves the use of macroquantitative statistical methods. Compared with other research programs, the method occupies a particularly prominent space in this perspective. *Georg Wenzelburger and Carsten Jensen* (2022) focus their critical assessment of this perspective on the macroquantitative method of comparative public policy. The authors identify several major weaknesses in the predominant use of macroquantitative methods in policy research. One of them is the use of covariance on the state level of variables describing socioeconomic, political, or institutional factors on the one hand and variables describing policy outcomes on the other hand. Comparative public policy could benefit from a closer look at policy processes, not just their conditions and outcomes. A second challenge is the often-oversimplified assumption of preferences of corporate actors. Here, the paper reminds us of the importance of the various actor-centered perspectives, including actor-centered institutionalism (Scharpf 1997) as well as ACF, MSF, and PAF, which are discussed in this special issue. Related to this is the claim to focus more on a systematic selection of cases and to focus on process instead of output when establishing relationships empirically.

Policy Feedback Theory (PFT) was originally based on questions and concepts of historical institutionalism. It studies the influence of policies on the subsequent policy agenda and problem definition. In the broader contemporary perspective, it examines policy feedback effects on later policies in different ways, including the political influence of interest groups and structures of government (Béland and Schlager 2019; Larsen 2019; Mettler and Sorelle 2018). In his contribution to this special issue, *Marius R. Busemeyer* (2022) emphasizes that these perspectives are also rooted in the United States. When transferring this perspective to the policy process of European countries and especially to the German context, the normative implications of the original PFT must be taken into account. One central argument of the paper concerns the different function and normative assessment of interest groups in U.S. pluralism compared to European corporatism. In countries with pluralistic interest intermediation, it is an understandable perspective to examine the emergence of special interest organizations as a possible problematic consequence of policies. In corporatism, by contrast, interest groups are not bound by individual policies but take on broad roles in policy formulation, implementation, and legitimation. The strengthening of interest groups can therefore often be viewed positively here, as it can enforce the responsiveness of the policy process. A second central argument of Busemeyer concerns the role of political parties and, among other things, the different current crises of party systems in the United States and Europe. In U.S. presidentialism, the current polarization and rise in importance of political parties is a symptom of crisis. In parliamentarism, on the other hand, parties must be seen as important legitimate elements of policy feedback. Here, the loss of importance of established parties and the strengthening of populist movements are more likely to be seen as problematic feedback.

Different challenges can be found by using the concept of policy styles that emerged during Margaret Thatcher’s government in the United Kingdom (Richardson 1982). It provided an important point of reference for comparative policy research, especially in Europe, by using the categories of anticipatory vs. reactive and consensus vs. imposition to compare longer-term features of policy processes in Western Europe. Although the original concept was developed only for established democracies, Howlett and Tosun have proposed a more general typology that can also be applied to autocratic political systems. Accordingly, policy styles should be distinguished depending on the key actors of the policy-making process on the one hand and the general state–society relationships on the other. First applications of this perspective show that policy styles need not be stable (Howlett and Tosun 2019). But what leads to changes over time? In their contribution to this special issue, *Jale Tosun, Maria Tullia Galanti, and Michael Howlett *(2022) examine the influence of political leadership on policy styles, using the data of the Sustainable Governance Indicators that cover 41 European Union and Organization for Economic Cooperation and Development countries (Bertelsmann Stiftung 2020). The authors select the four cases of Malta, Italy, Ireland, and Poland. The Maltese policy style changed because of an external event and then became entrenched, which also occurred for Ireland, where the austerity regime reduced the role of societal consultation. The policy style in Italy remained stable over time but changed only when the populist government of Giuseppe Conte (2018–2021) came into power. In Poland, political leadership has weakened participatory elements since 2017.

Like the concept of policy styles, the origins of evidence-based policy-making (EBP) are linked to British politics. Evidence-based policy-making emerged in relation to the idea of third-way politics by Tony Blair’s government from 1997 on (Giddens 1998). The concept claims to justify policies less ideologically and to derive them from scientific knowledge instead. However, this requires not only generating scientific knowledge but also selecting and introducing it into the policy process. To this end, different systems of scientific policy advice have been created in different countries, policy fields, and situations. In their contribution to this special issue, *Susanne Hadorn, Fritz Sager, Céline Mavrot, Anna Malandrino, and Jörn Ege* (2022) compare the advisory bodies in Germany, Switzerland, and Italy that were used to deal with the COVID-19 pandemic. The crisis was confronted by quite opposite bodies of scientific advice in the three countries: In Germany, there is a tradition of long-standing advisory bodies for the line ministries, which are usually based only on the expertise of a few selected scientific disciplines (Bandelow et al. 2013). Switzerland primarily uses short-term mandates with external scientists for policy formulation, with a strong focus on policy evaluation. The Italian advisory system is characterized by the authors as underdeveloped and politicized; Italian pandemic policy was characterized by uncertainty and conflict among different levels (Malandrino and Demichelis 2020). The authors evaluate these three systems’ performance during the pandemic by categorizing them according to their foundation on expertise and evidence and assessing the salience, credibility, and representativeness that they convey. Against this background, the authors formulate expectations for the functioning of policy advisory systems during the crisis. These expectations are only partially met. It becomes apparent that situational factors in addition to the established structures of EBP must be considered in times of crisis. Nevertheless, the general systems of policy advice also have an impact on the opportunities for EBP during the crisis: Among others, the development of institutionalized relationships between specialized scientists and decision-makers is an important basis for quickly bringing timely and relevant expertise into the decision-making process.

## Conclusion: Public Policy Is Still at the Beginning of Its Journey Through the World

The contributions to this special issue demonstrate the extent to which public policy research has become a global endeavor. Despite the differences in political systems, political cultures, educational systems, administrative systems, and forms of policy deliberation, there are commonalities that can be captured through the perspectives presented here. In all countries, policy processes are characterized by conflict and the struggle for broad support among stakeholders. Therefore, examining the policy-related goals, strategies, and alliances of actors can regularly contribute to the understanding of policies. Policy actors always act on the basis of some form of bounded rationality. Here, the different approaches allow us to capture different aspects. These range from beliefs to social identities to ambiguity. In addition to the respective subsystem actors, external and situational conditions must always be considered. Other levels and policy fields can influence policy decisions in subsystems. The specific forms of influence, in turn, depend on the particularities of the case under study.

In each case, the approaches complement each other in highlighting certain aspects of the policy process. In view of the still prevailing regional imbalance in the origin of approaches, this results in a challenge that could not yet be considered here: Specific features of countries and regions that have received little attention to date may remain unaddressed. This imbalance is already observable in the transfer of American approaches to Europe. The differences in party systems, for example, needed to be considered before the approaches could be applied outside the U.S. context. But there are also institutions and traditions that are specific for only one or a few countries. Think of the particular role the rule of law plays in Germany, for example. In France, policy processes are populated by elites with particular biographies, and in Switzerland, direct democracy shapes policy-making in an outstanding way uncommon elsewhere. All this has led to the emergence of unique perspectives and approaches in national debates outside the Anglo-Saxon world. This development is even more true for policy research in Asia, South America, and Africa. There are many peculiarities of social, economic, and political systems of southern countries.

In this special issue, we have discussed what to consider when transferring approaches from the United States to other countries. A further, future step may be to reverse the theory transfer as well. For example, states such as Iran can be discussed to illustrate how an interplay between religious and secular institutions can affect the policy process. Theories that emerge from these observations can then, in turn, also contribute to analyzing hitherto little-noticed peculiarities of the policy process in Europe or North America. It will be an exciting challenge for future policy research to use the two-way international transfer of theories and methods to generate more knowledge of and for the policy process. How fruitful a reverse transfer of public policy knowledge from the South to the North can be depends on many factors. Well-equipped research institutes in the field do not exist everywhere yet. Most importantly, policy research often faces challenges of political constraints: It is not desirable in all countries for scholars from around the world to conduct interviews with political actors and to trace policy processes. Against this background, it is necessary to consider the ontological diversity of policy processes as well as the epistemological challenge of obtaining data and limiting the possibilities of publication.

Compared to this challenge, the linguistic diversity in the transfer of approaches seems to be a smaller problem. However, the introductory reference to the conceptual diversity in naming the subject in different languages shows that there is more involved in the use of English as the language of science than a simple translation into a generally understandable form: Especially in politics, linguistic subtleties, specific metaphors, historical conceptual understandings, and other challenges take on significance that is lost when translated into English.

This introduction shows that we are still at the very beginning of the internationalization of policy research. Against the background of these restrictions, this special issue aims to contribute to the currently growing discussion on the scope and fit of theoretical perspectives in policy research. At the same time, it picks up where the now legendary PVS special issue of 1993 left off. The SI editors hope to have a similarly lasting impact on the professional discussion, not only in Germany but worldwide.

Should this goal be achieved, thanks are due first and foremost to the authors, who almost without exception have agreed quickly and worked in a disciplined manner. Also, the PVS editorial office and especially Christian Adam, as supervising editor responsible for the review process, have been very supportive. Finally, we would like to thank the reviewers, who provided constructive comments on all contributions and in each case made the rapid realization of this project possible.
